# Age-Dependent Degeneration of Mature Dentate Gyrus Granule Cells Following NMDA Receptor Ablation

**DOI:** 10.3389/fnmol.2015.00087

**Published:** 2016-01-12

**Authors:** Yasuhito Watanabe, Michaela K. Müller, Jakob von Engelhardt, Rolf Sprengel, Peter H. Seeburg, Hannah Monyer

**Affiliations:** ^1^Department of Clinical Neurobiology, University Hospital and German Cancer Research Center HeidelbergHeidelberg, Germany; ^2^Synaptic Signalling and Neurodegeneration, German Center for Neurodegenerative DiseasesBonn, Germany; ^3^Synaptic Signalling and Neurodegeneration, German Cancer Research Center HeidelbergHeidelberg, Germany; ^4^Department of Molecular Neurobiology, Max Planck Institute for Medical ResearchHeidelberg, Germany

**Keywords:** NMDA receptors, aging, neurodegeneration, hippocampus, dentate gyrus, mouse

## Abstract

*N*-methyl-*D*-aspartate receptors (NMDARs) in all hippocampal areas play an essential role in distinct processes of memory formation as well as in sustaining cell survival of postnatally generated neurons in the dentate gyrus (DG). In contrast to the beneficial effects, over-activation of NMDARs has been implicated in many acute and chronic neurological diseases, reason why therapeutic approaches and clinical trials involving receptor blockade have been envisaged for decades. Here we employed genetically engineered mice to study the long-term effect of NMDAR ablation on selective hippocampal neuronal populations. Ablation of either GluN1 or GluN2B causes degeneration of the DG. The neuronal demise affects mature neurons specifically in the dorsal DG and is NMDAR subunit-dependent. Most importantly, the degenerative process exacerbates with increasing age of the animals. These results lead us to conclude that mature granule cells in the dorsal DG undergo neurodegeneration following NMDAR ablation in aged mouse. Thus, caution needs to be exerted when considering long-term administration of NMDAR antagonists for therapeutic purposes.

## Introduction

The dentate gyrus (DG) in the hippocampus has been the focus of numerous studies for two main reasons. Firstly, the DG is a major station in the trisynaptic pathway that conveys input from the cortex to the hippocampus. DG granule cells excite CA3 neurons, which in turn project to CA1 neurons. All hippocampal subregions have been much investigated in the context of distinct forms of plasticity, learning, and memory (e.g., McHugh et al., 2007; Niewoehner et al., 2007; Gu et al., 2012; Nakashiba et al., 2012). Secondly, the DG is one of the two principal neurogenic niches in the postnatal brain, and has been therefore frequently studied in the context of neurogenesis (reviewed in Jessberger and Gage, 2014). Some studies addressed questions that link these two research areas. Thus, young DG granule cells support pattern separation, whereas mature granule cells facilitate pattern completion (Nakashiba et al., 2012). The DG comprises a dorsal and a ventral part that can be distinguished on anatomical grounds, and also based on distinct functions that the two areas support. Thus, the dorsal DG is involved in cognitive functions such as spatial memory, whilst the ventral DG supports brain functions related to stress, emotion, and affect (reviewed in Fanselow and Dong, 2010). Several decades of pharmacological and genetic research have taught us that *N*-methyl-*D*-aspartate receptors (NMDARs) are intricately linked to hippocampus-dependent memory (reviewed in Bannerman et al., 2014). Mouse genetics was fundamental to correlate subregions of the hippocampus, including the DG, with distinct functions supporting spatial memory (McHugh et al., 2007; Niewoehner et al., 2007). Thus, NMDARs in the DG are required for pattern separation (McHugh et al., 2007) and spatial working memory (Niewoehner et al., 2007). Furthermore, studies based on mouse mutants were instrumental in helping delineate the differential functions of NMDAR subunits. DG granule cells, like most neurons in the hippocampus, harbor NMDARs built from the obligatory GluN1 subunit (encoded by *Grin1*) and the developmentally regulated GluN2A and GluN2B subunits (encoded by *Grin2a* and *Grin2b*, respectively; Monyer et al., 1994; Rodenas-Ruano et al., 2012; Paoletti et al., 2013). NMDARs have been much discussed with respect to their function in postnatal neurogenesis. Thus, NMDARs in postnatally generated DG granule cells are a prerequisite for the survival of newborn neurons (Tashiro et al., 2006).

In mature neurons, over-activation or suppression of NMDARs are detrimental for cell function and survival. Thus, enhanced receptor activity as it occurs in many pathophysiological neurological diseases, e.g., stroke, epilepsy, induces excitotoxic cell death (reviewed in Choi, 1992; Paoletti et al., 2013). Conversely, pharmacological blockade of NMDARs for hours and days also has an adverse effect, as it induces cellular vacuolization in pyramidal neurons in many cortical brain areas (e.g., Ikonomidou et al., 1999). The effect of prolonged or even permanent NMDAR blockade on the survival of mature neurons has not yet been tested systematically. Moreover, a potential role of NMDARs for cell survival in aged animals has not been investigated. This is surprising, given that the NMDAR has been a key candidate when considering therapeutic strategies for stroke.

Here we took advantage of genetically modified mice and addressed the question whether the survival of mature and aged neurons depends on NMDARs. We demonstrate that in the hippocampus, mature neurons in the DG, but not in the CA1 region, require intact NMDAR function for survival.

## Materials and Methods

### Animals

Animals were housed and handled according to the respected animal welfare guidelines and rules of the Max Planck Society and the Germany government animal welfare office in Karlsruhe, Germany. The following genetically modified male and female C57Bl/6N mice were used in this study: DG and CA1 selective *Grin1* knockout (*Grin1*^Δ*DGCA1*^) mice (Bannerman et al., 2012), DG selective *Grin1* knockout (*Grin1*^Δ*DG*^, previously called *NR1*^Δ*DG*^) mice (Niewoehner et al., 2007), *Grin2a* knockout (*Grin2a^-/-^*, previously called homozygous *GluR𝜀1* mutant) mice (Sakimura et al., 1995), and DG and CA1 selective *Grin2b* knockout (*Grin2b*^Δ*DGCA1*^, previously called *NR2B* ^Δ*HPC*^) mice (von Engelhardt et al., 2008).

*Grin1*^Δ*DGCA1*^ mice are homozygous for the floxed *Grin1* and carry two transgenes, *Tg^LC1^* and *Tg^CN12^*, which enable doxycycline-sensitive, Cre-mediated gene ablation in CA1 and DG excitatory neurons and piriform cortex in the adult brain by use of a *CamKIIa/Grin2c* hybrid promoter (Bannerman et al., 2012). *Grin1*^Δ*DG*^ mice are homozygous for *Grin1* and carry the transgenes *Tg^LC1^* and *Tg^CN10-itTA^*, which are the same transgenes as in *Tg^CN12^*, but yield differential expression in the two transgenic mouse lines. In *Grin1*^Δ*DG*^ mice, Cre is specifically expressed in the DG granule cells and some CA1 pyramidal neurons (Niewoehner et al., 2007) which express calbindin (CB; data not shown). *Grin2b*^Δ*DGCA1*^ mice are homozygous for the floxed *Grin2b* and carry *Tg^LC1^* and *Tg^CN12^*. For the selective expression of Cre in *Grin1*^Δ*DGCA1*^, *Grin1*^Δ*DG*^ and *Grin2b*^Δ*DGCA1*^ mice, doxycycline was given via the drinking water to pregnant mice to suppress Cre expression of the offspring during embryonic development, and was withdrawn after birth (Bannerman et al., 2012). Doxycycline treatment was performed under the license 35-9185.81/G71/10 of the governmental council in the Karlsruhe, Germany. In *Grin1*^Δ*DGCA1*^ mice, Cre expression becomes detectable as early as postnatal day P28 (Bannerman et al., 2012). Control mice (i.e., mice with no Cre expression) to be compared with *Grin1*^Δ*DGCA1*^ and *Grin1*^Δ*DG*^ mice were homozygous for floxed *Grin1* and carried one or none of the transgenes. Control mice for the *Grin2a* knockout mice are wild-type mice. Control mice for *Grin2b*^Δ*DGCA1*^ mice were homozygous for floxed *Grin2b* and carried one or none of the transgene. As control mice for Cre expressing mice (carrying only *Tg^LC1^* and *Tg^CN12^* but no floxed gene) we used either wild-type mice or carriers of one of the transgenes. Following mice are available at EMMA (https://www.infrafrontier.eu) with respective EMMA ID: Floxed *Grin1* mice (EM: 09220), mice carrying *Tg^LC1^* and *Tg^CN12^* transgenes (EM: 09256), and mice carrying *Tg^CN10-itTA^* transgene (EM: 09255). All animal experiments were performed according to the regulations of Heidelberg University/German Cancer Research Center/Max Planck Institute.

### Immunohistochemistry

Mice were perfused with phosphate buffered saline (PBS, pH 7.4) followed by 4% paraformaldehyde (PFA). Brains were removed and postfixed with 2% PFA at 4°C overnight. Coronal sections were prepared at 50 μm using a vibratome. Sections were blocked and permeabilized with 5% bovine serum albumin (BSA) in PBS containing 0.5% Triton X-100, incubated with primary antibodies overnight at 4°C, then incubated with appropriate secondary antibodies. Primary and secondary antibodies were diluted in PBS containing 0.2% Triton X-100 before incubation. Sections were washed three times with PBS after every antibody incubation step. Sections were counterstained with DAPI (Invitrogen, Carlsbad, CA, USA) after the secondary antibody incubation step. For GluN1 staining, before blocking the sections, antigen retrieval was performed as follows. Sections were immersed in 10 mM sodium citrate (pH 6.0) and heated in a microwave oven for 5 min at 650 W twice with a 5 min break in between, kept for 20 min at room temperature, and washed twice with PBS. The following primary antibodies were used: rabbit active caspase-3 (catalog # AF835; 1:2000; polyclonal; R&D systems, Minneapolis, MN, USA), mouse CB D-28k (300; 1:2000; monoclonal; Swant, Switzerland), goat DCX (sc-8066; 1:500; polyclonal; Santa Cruz Biotechnology, Santa Cruz, CA, USA), goat Sox-2 (sc-17320; 1:500; polyclonal; Santa Cruz Biotechnology), mouse GFAP (G3893; 1:10000; monoclonal; Sigma–Aldrich, Saint Louis, MO, USA), rabbit S100β (721; 1:2000; polyclonal; Swant), rabbit NMDAR1 (for GluN1; AB9864R; 1:250, monoclonal; Millipore, Billerica, MA, USA), rabbit Cre [1:2000; polyclonal; a generous gift from Dr. Günther Schütz (Kellendonk et al., 1999)], mouse Mineralocorticoid receptor [6G1; 1:100; monoclonal; a generous gift from Dr. Elise P. Gomez-Sanchez (Gomez-Sanchez et al., 2006)].

### Nissl Staining

Sections were mounted on microscope slides, air dried, immersed in 0.1% thionin, washed with water, dehydrated in 70, 95, and 100% ethanol for several minutes, cleared in xylene for 5 min twice. The slides were subsequently covered with Eukitt mounting medium, and mounted with coverslips.

### Image Processing and Quantification

Fluorescent images were acquired using a LSM 700 confocal microscope (Zeiss, Oberkochen, Germany). A single focal plane picture of the whole DG area from one hemisphere was taken from each brain section using a tile scan function of the confocal microscope. Bright field images were acquired using a BX51W microscope (Olympus, Tokyo, Japan). All quantification analyses were done using ImageJ software. The DG thickness was measured from both dorsal/suprapyramidal blade and ventral/infrapyramidal blade. Care was taken to minimize variations in choosing sections in the rostrocaudal axis. Several, typically three, DG images from one animal were used for the analysis whenever possible. Measured values were averaged to create one representative value for each animal. To calculate DG thickness, a part of the DG with uniform thickness was selected as rectangular area of interest, and the area was then divided by the base length of the rectangular area. Dorsal DG thickness was calculated from the proximal areas to the crest where the two blades connect. Ventral DG thickness was calculated from the most ventral area close to the end of the blade. Degenerating cells were counted using the green channel after staining with antibody against active caspase-3. A cell was considered DCX-positive when the signal outlined the shape of a cell body with processes. To confirm that active capsase-3 (stained in green fluorescence) is expressed in CB-positive (stained in red fluorescence) cells, images of active caspase-3 positive cells were taken when there was no auto-fluorescence signal from degenerating cells in an unstained (far-red fluorescence) channel. Degenerating cells were counted as follows. The DG area to be examined was selected with selection tools. Putative degenerating cells were selected by MaxEntropy method of the autothreshold function, and signals bigger than 3 μm^2^ were counted using “Analyze particles” function. We confirmed that this counting method matches manual counting. This method enabled us to quantify degenerating cells more objectively than manual counting. Except for degenerating cells, all other fluorescently labeled cells were counted manually using cell counter function of ImageJ. Three to six images of the DG were used for counting fluorescent positive cells, and the obtained values were pooled to obtain one value for each mouse.

### Statistical Analysis

Quantifications were carried out without referring to the genotype information. Differences or correlations between groups were examined using the statistical tests indicated in the figure legends. Statistical analyses were performed using R 3.1.3. Values were expressed as mean ± standard deviation unless otherwise mentioned in the figure legends. Values of *p* < 0.05 were considered statically significant (^∗^*p* < 0.05, ^∗∗^*p* < 0.005, ^∗∗∗^*p* < 0.0005).

## Results

### GluN1 Ablation Causes Progressive Degeneration of Granule Cells in the Dorsal DG

**FIGURE 1:**
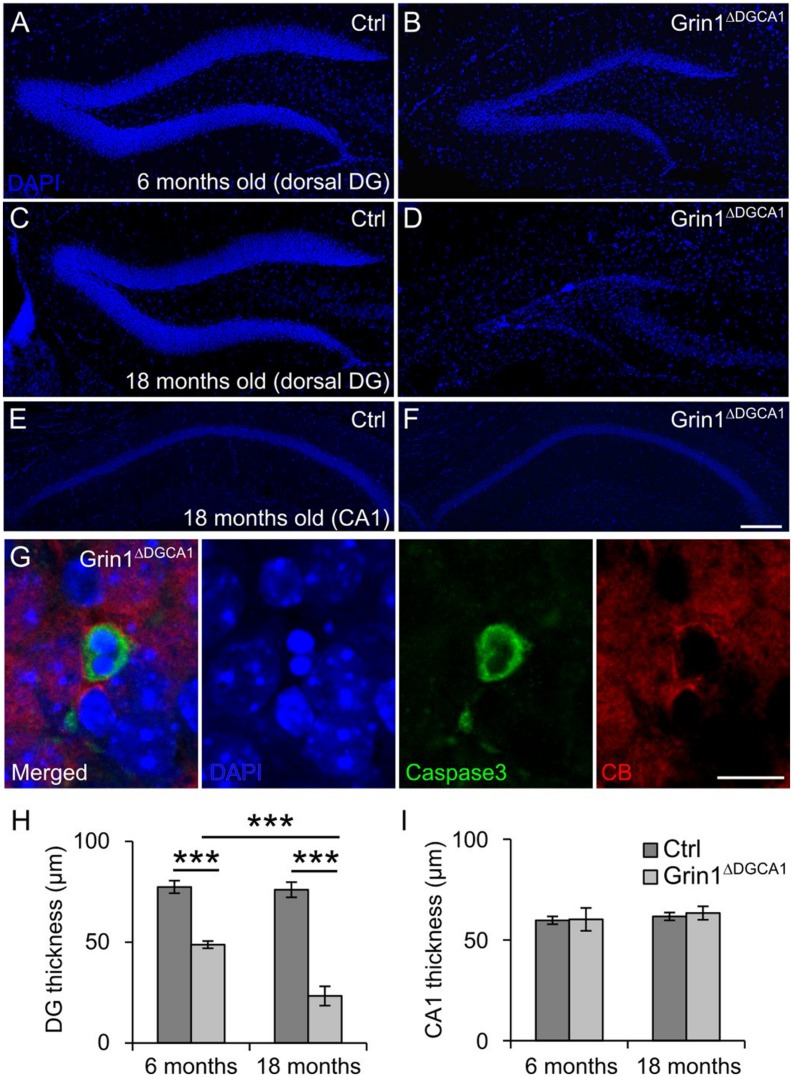
**GluN1 deletion causes age-dependent neurodegeneration in the dorsal dentate gyrus (DG). (A)** DAPI staining illustrating a section of a dorsal DG from a 6 months old control (Ctrl) mouse. **(B)** DAPI staining of a dorsal DG from a 6 months old *Grin1*^Δ*DGCA1*^ mouse. Note the thinning of the dentate granule cell layer. **(C)** DAPI staining illustrating a section of a dorsal DG from an 18 months old control mouse. **(D)** DAPI staining of a dorsal DG from an 18 months old *Grin1*^Δ*DGCA1*^ mouse. Note the thinning of the dentate granule cell layer following profound neurodegeneration. **(E)** DAPI staining illustrating a section of a dorsal CA1 from an 18 months old control mouse. **(F)** DAPI staining of a dorsal DG from an 18 months old *Grin1*^Δ*DGCA1*^ mouse. There is no difference between the two genotypes. **(G)** Representative image from the DG showing an apoptotic, i.e., caspase-3-positive, CB-positive neuron in a 6 months old *Grin1*^Δ*DGCA1*^ mouse. The section is counterstained with DAPI. **(H)** Quantitative evaluation of dorsal DG thickness in 6 and 18 months old control and *Grin1*^Δ*DGCA1*^ mice. The thickness of the DG was analyzed by two-factor ANOVA, followed by pairwise comparison test with *p*-value modification by Holm’s method, ^∗∗∗^*p* < 0.0005. The DG is thinner in *Grin1*^Δ*DGCA1*^ mice [*F*(1,16) = 662.39, *p* < 0.0005], and the difference increases with aging [*F*(1,16) = 59.92, *p* < 0.0005]. There is a significant interaction between genotype and age [*F*(1,16) = 53.99, *p* < 0.005]. **(I)** Quantitative evaluation of CA1 thickness in the dorsal hippocampus in 6 and 18 months old control and *Grin1*^Δ*DGCA1*^ mice. There is no difference in the thickness of the CA1 layer (two-factor ANOVA, no significant effect of genotype [*F*(1,16) = 0.594], no significant effect of age [*F*(1,16) = 2.793], and no significant interaction between age and genotype [*F*(1,16) = 0.168]. Scale bars, in **(F)**, which applies to **(A–E)**, and **(G)**, 200 and 10 μm, respectively. Five and four mice for 6 months old control and *Grin1*^Δ*DGCA1*^, and six and five mice for 18 months old control and *Grin1*^Δ*DGCA1*^ were used, respectively.

In 6 months old *Grin1*^Δ*DGCA1*^ mice, in which *Grin1* is deleted in both the DG and CA1, we found a reduced thickness of the DG granule cell layer (**Figures 1A,B,H**). This phenotype could be readily detected by eye after DAPI staining, and was more pronounced in 18 months old mice (**Figures 1C,D,H**). In contrast, there was no apparent abnormality in the CA1 region in 18 months old *Grin1*^Δ*DGCA1*^ mice (**Figures 1E,F,I**). Expression of active caspase-3 in CB-positive granule cells was indicative of mature granule cell demise (**Figure 1G**). The alteration in the granule cell layer was restricted to the dorsal DG. In 18 months old mice, the ventral DG in *Grin1*^Δ*DGCA1*^ mice was comparable to that of controls (Supplementary Figures S1A–C). We confirmed GluN1 ablation in the CA1 region and in both the dorsal and ventral DG of 6 months old *Grin1*^Δ*DGCA1*^ mice by immunohistochemistry (Supplementary Figures S1D–G). Neuronal degeneration did not result from Cre expression itself, but was a consequence of *Grin1* deletion via Cre-mediated recombination, as the DG was normal in Cre expressing mice with wild-type *Grin1* alleles (Supplementary Figure S2). Thus, ablation of NMDARs in CA1 and DG causes selective degeneration of granule cells in the dorsal DG.

### Neuronal Degeneration in *Grin1*^Δ*DGCA1*^ Mice Affects Mature DG Granule Cells

**FIGURE 2:**
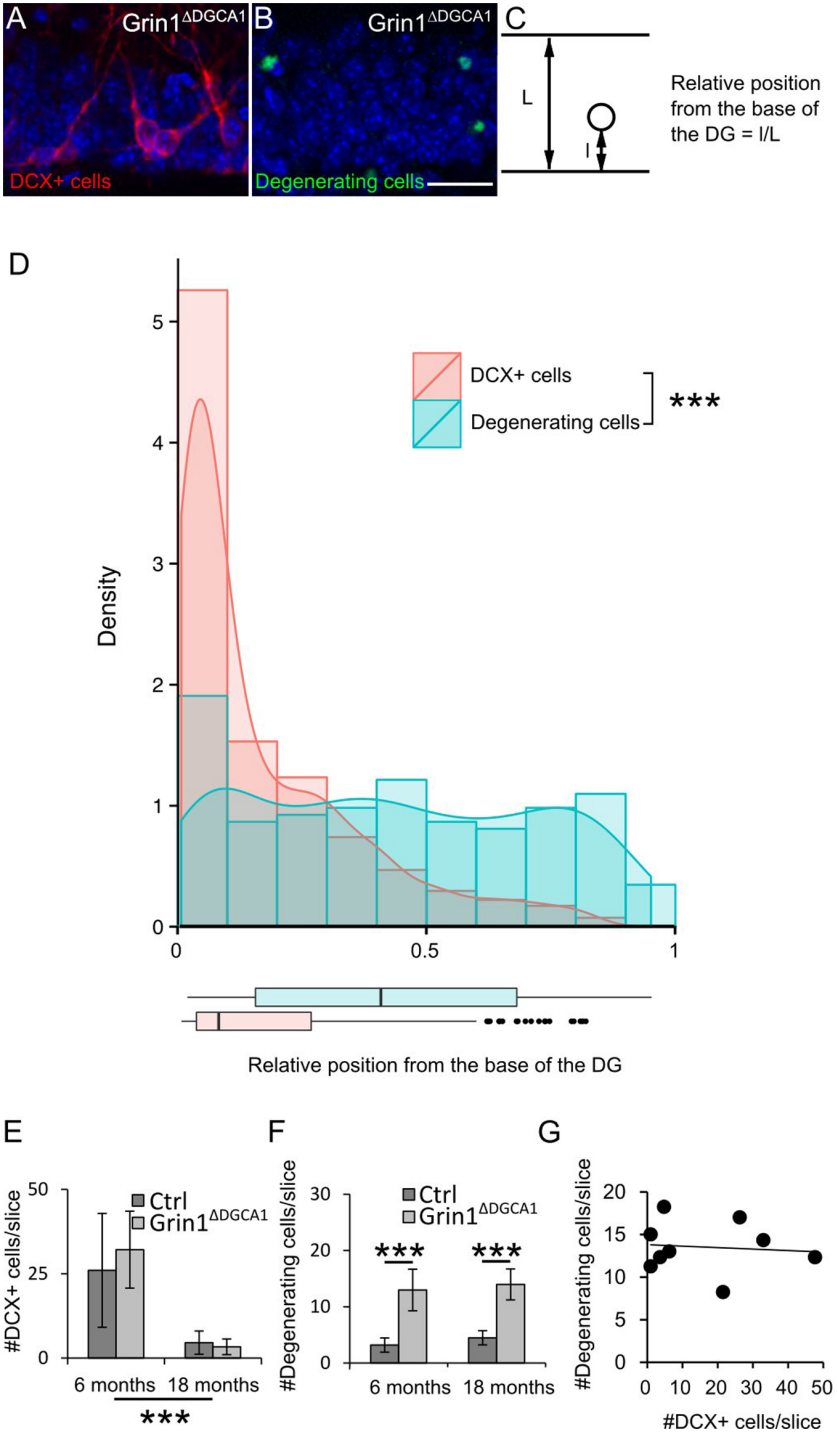
***N*-methyl-*D*-aspartate receptor (NMDAR) depletion in *Grin1*^Δ*DGCA1*^ mice causes age-dependent degeneration of mature DG granule cells. (A)** DAPI stained section (blue) from a 6 months old *Grin1*^Δ*DGCA1*^ mouse brain showing DCX-positive (abbreviated DCX+ in the figure) neurons (red) located in the inner DG granule cell layer. **(B)** DAPI stained section (blue) from a 6 months old *Grin1*^Δ*DGCA1*^ mouse with degenerating neurons (green) dispersed throughout the layer. **(C)** Diagram indicating the parameters that were used to calculate the relative position of individual DG granule cells. **(D)** Histogram, density plot (curved line), and boxplot (bottom) of the relative position of DCX-positive cells (pink) and degenerating cells (blue) showing the differential distribution within the DG granule cell layer (significant difference in both Kolmogorov–Smirnov Tests and Mann–Whitney–Wilcoxon Test, ^∗∗∗^*p* < 0.0005). Whiskers extending from the boxes in the boxplot indicate the maximum and minimum value within the 1.5-fold of the interquartile range from the higher (75th percentile) and the lower quartile (25th percentile). Dots in the boxplot are outliers. This analysis comprised 405 DCX-positive cells and 173 degenerating cells. **(E)** The number of DCX-positive cells in the DG decreases with age both in control and *Grin1*^Δ*DGCA1*^ mice [two-factor ANOVA, *F*(1,16) = 30.090, *p* < 0.00005]. There is neither a significant difference between genotypes [*F*(1,16) = 0.653], nor a significant interaction between age and genotype [*F*(1,16) = 0.423]. **(F)** The number of degenerating neurons in the DG is higher in *Grin1*^Δ*DGCA1*^ mice [two-factor ANOVA, *F*(1,16) = 85.593, *p* < 0.00005, followed by *post hoc* analysis with Holm’s *p*-value modification, ^∗∗∗^*p* < 0.0005]. There is neither an effect of age [*F*(1,16) = 0.247, ns], nor a significant interaction between age and genotype [*F*(1,16) = 0.888]. **(G)** There is no correlation between the number of degenerating and newborn neurons in the DG of *Grin1*^Δ*DGCA1*^ mice (Spearman’s rank correlation coefficient test). The same animals were used as in **Figure 1**. Scale bar in **(B)**, 25 μm.

To corroborate that NMDAR deletion causes cell death of mature DG granule cells, we compared the distribution of immature doublecortin (DCX) expressing cells (**Figure 2A**) and of degenerating cells visualized by active caspase-3 immunostaining (**Figure 2B**) within the DG granule cell layer (**Figure 2C**). If degeneration affected primarily immature DG granule cells, degenerating cells should be confined to the innermost rim of the DG granule cell layer that harbors DCX expressing neurons. This, however, was not the case. Degenerating cells were found throughout the entire DG granule cell layer (**Figure 2D**). We next examined whether there was a correlation between the number of immature and degenerating DG granule cells. Since the number of immature DG granule cells decreases with age, both populations would have to decrease in parallel, if degenerating cells were primarily immature DG granule cells. However, while the number of immature DG granule cells decreased with age both in control and *Grin1*^Δ*DGCA1*^ mice (**Figure 2E**), the number of degenerating cells was higher in *Grin1*^Δ*DGCA1*^ mice than in controls both at 6 and 18 months of age (**Figure 2F**). Furthermore, in *Grin1*^Δ*DGCA1*^ mice, there was no correlation between the number of immature DG granule cells and the number of degenerating cells, suggesting that degenerating cells were not immature cells (**Figure 2G**). Together these results indicate that degenerating DG granule cells are primarily mature DG granule cells.

### NMDAR Dependent Survival is a Cell Type-Specific Property of Mature DG Granule Cells

**FIGURE 3:**
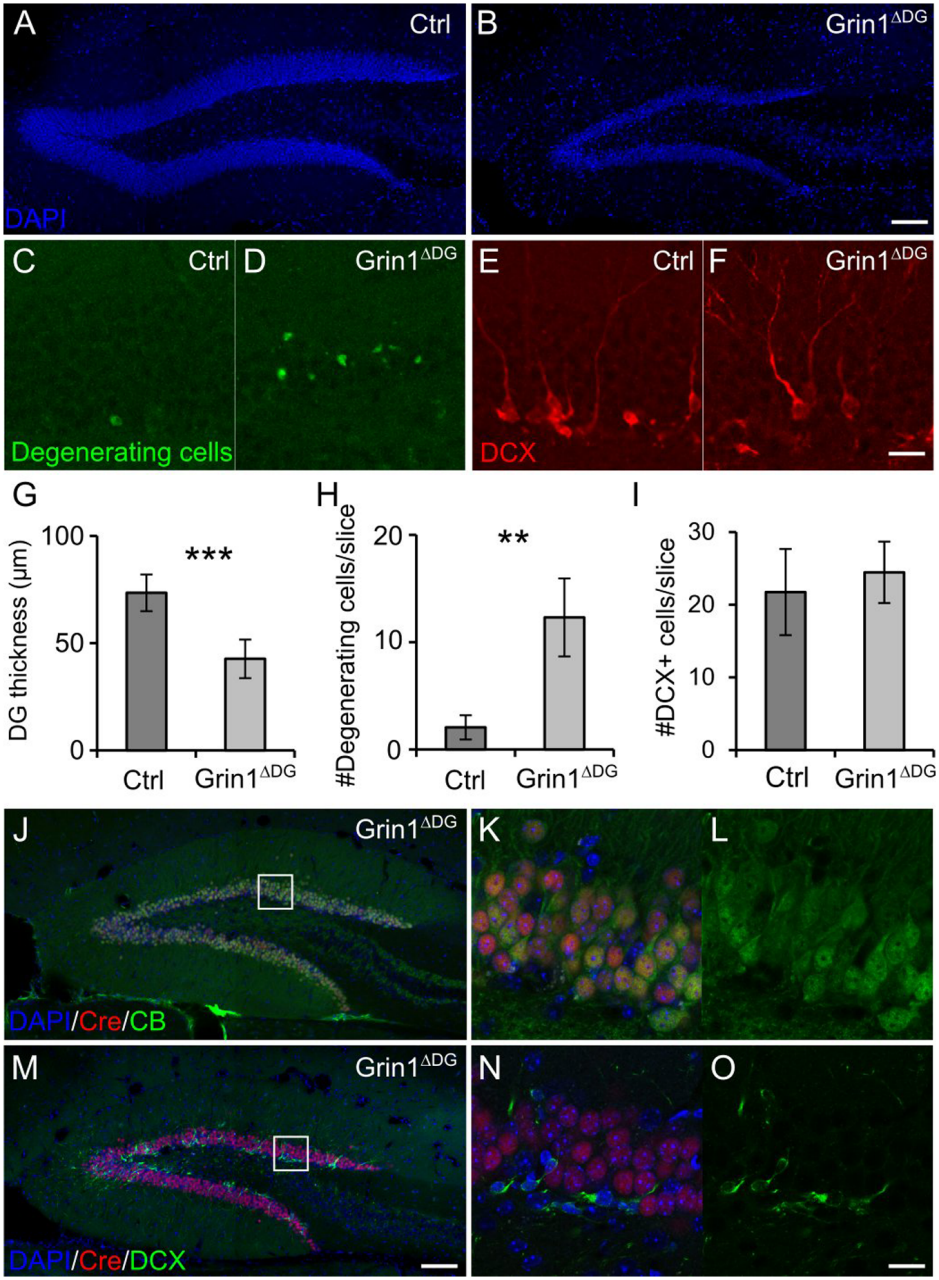
**DG selective deletion of GluN1 in *Grin1*^Δ*DG*^ recapitulates the phenotype observed in *Grin1*^Δ*DGCA1*^ mice. (A)** DAPI stained section showing the DG of a 6 months old control mouse. **(B)** DAPI stained section from a 6 months old *Grin1*^Δ*DG*^ mouse brain showing thinning of the DG. **(C–F)** Degenerating cells [green in **(C,D)**] and DCX-positive cells [red in **(E,F)**] in the DG of a 6 months old control and *Grin1*^Δ*DG*^ mouse. **(G)** DG thickness is reduced in *Grin1*^Δ*DG*^ mice (Welch’s *t*-test, ^∗∗∗^*p* < 0.0005). **(H)** The number of degenerating cells is increased in the DG of *Grin1*^Δ*DG*^ mice (Welch’s *t*-test, ^∗∗^*p* < 0.005). **(I)** The number of DCX-positive immature cells is comparable between genotypes (Welch’s *t*-test). **(J–O)** In the DG of *Grin1*^Δ*DG*^ mice, Cre (red) is expressed in CB-positive [green in **(J–L)**] mature neurons, but not in DCX-positive [green in **(M–O)**] immature neurons. Right panels **(K,L,N,O)** show a magnified view of the indicated area [white boxes in **(J,M)**]. Scale bars in **(B,F,M,O)**, 100, 20, 100, and 20 μm, respectively. Five and seven mice were used for control and *Grin1*^Δ*DG*^, respectively.

In *Grin1*^Δ*DGCA1*^ mice, DG granule cell degeneration might be a complex phenotype that results from changed interactions between CA1 and DG network activities, and might reflect, at least in part, altered output activity from CA1 neurons, in which NMDARs were also deleted. To directly test whether the observed degeneration of mature DG granule cells following GluN1 depletion is indicative of a specific DG granule cell vulnerability, we investigated the effect of NMDAR depletion in genetically modified mice (*Grin1*^Δ*DG*^), in which the manipulation was restricted to the DG. Indeed, a reduction in the thickness of the dentate granule cell layer with degenerating cells was seen also in 6 months old *Grin1*^Δ*DG*^ mice (**Figures 3A–D,G,H**), indicating that adult granule cells require NMDAR-mediated activity for their survival.

Similarly to *Grin1*^Δ*DGCA1*^ mice, also in *Grin1*^Δ*DG*^ mice the number of immature DG granule cells was not reduced (**Figures 3E,F,I**), suggesting that GluN1 depletion was restricted to mature DG granule cells. Hence we determined Cre expression in mature and immature DG granule cells, and found that indeed, Cre expression was detected in CB-positive, but not DCX-positive neurons both in *Grin1*^Δ*DG*^ (**Figures 3J–O**) and in *Grin1*^Δ*DGCA1*^ mice (data not shown). Accordingly, GluN1 expression was present in immature DG granule cells (Supplementary Figures S3A–D). Together, these data provide evidence that GluN1 ablation was restricted to mature DG granule cells.

We wondered whether DG granule cell degeneration following NMDAR ablation was noticeable already at earlier developmental stages, and thus investigated 2 months old *Grin1*^Δ*DG*^ mice. Despite of the GluN1 ablation, the number of immature and degenerating DG granule cells, and the overall thickness of the granule cell layer were comparable between control and mutant mice (Supplementary Figures S3E–I). It thus appears that the phenotype of DG granule cell degeneration following NMDAR ablation worsens in an age-dependent fashion.

Next we examined whether the genetic manipulation altered the number of stem cells in the DG of 6 months old *Grin1*^Δ*DG*^ mice. The number of stem cells, i.e., GFAP/Sox2 positive cells, was not different between the two genotypes. The increased number of GFAP/Sox2/S100β positive cells in *Grin1*^Δ*DG*^ mice (Supplementary Figure S4), reflects enhanced proliferation of astrocytes as often reported in brain areas with neurodegeneration. Hence NMDAR depletion in mature DG granule cells does not affect stem cell proliferation.

### Survival of Mature DG Granule Cells Requires GluN2B Expression

**FIGURE 4:**
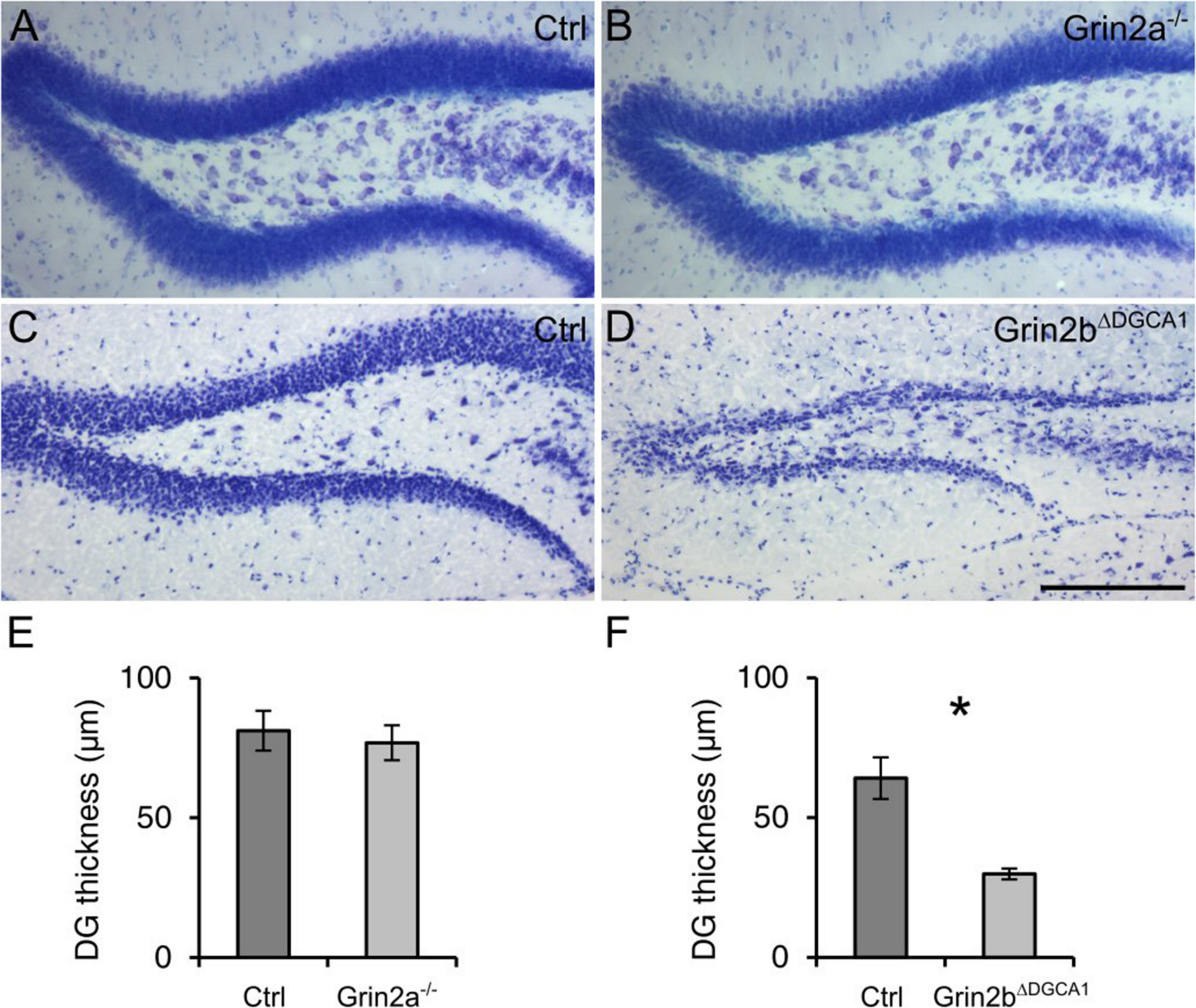
**Absence of GluN2B-, but not GluN2A-containing NMDARs causes age-associated neurodegeneration of granule cells in the dorsal DG. (A)** Nissl staining of the DG from an 18 months old control mouse. **(B)** Nissl staining of the DG from an 18 months old *Grin2a^-/-^* mouse. **(C)** Nissl staining of the DG from an 18 months old control mouse. **(D)** Nissl staining of the DG from an 18 months old control mouse (*Grin2b*^Δ*DGCA1*^). **(E)** Quantitative evaluation of the granule cell layer thickness in the DG of control and *Grin2a^-/-^* mice (Student’s *t*-test, ns). **(F)** Quantitative evaluation of the granule cell layer thickness in the DG of control and *Grin2b*^Δ*DGCA1*^ mice (Welch’s *t*-test, ^∗^*p* < 0.05). Scale bar in **(D)**, 200 μm. Four and three mice for control and *Grin2a^-/-^*, and three and three mice for control and *Grin2b*^Δ*DGCA1*^ were used, respectively.

Finally, to examine the composition of the NMDARs involved in the degeneration of DG granule cells, we investigated the survival of mature DG granule cells in mice with either GluN2A or GluN2B receptor ablation. To this end we took recourse to 18 months old *Grin2a^-/-^* and *Grin2b*^Δ*DGCA1*^ mice. In *Grin2a^-/-^* mice, the overall appearance and thickness of the DG granule cell layer was not different from that in control mice (**Figures 4A,B,E**). In contrast, the DG of *Grin2b*^Δ*DGCA1*^ mice was reminiscent of the above-described phenotype in *Grin1*^Δ*DGCA1*^ and *Grin1*^Δ*DG*^ mice (**Figures 4C,D,F**). These data demonstrate that GluN2B-containing NMDARs support the survival of aged dorsal DG granule cells.

## Discussion

Here we demonstrate that NMDAR ablation causes neurodegeneration of the DG. This phenotype derives from the demise of mature DG granule cells and not from altered neurogenesis for the following reasons: First, enhanced caspase-3 expression and degenerating neurons were detected in the outer DG granule cell layer in the mutant mice. Second, the number of stem cells in the DG did not differ between control and mutant mice. Third, neurogenesis in the DG is known to decrease with age. Thus, if NMDAR ablation affected newborn cells, one would expect a progressive age-dependent decline of degenerating neurons. This, however, was not the case. Fourth, GluN1 was depleted primarily in mature DG granule cells as evidenced both by Cre expression and GluN1 immunohistochemistry in *Grin1*^Δ*DG*^ mice. In addition, previous studies indicated that in several mouse mutants with reduced adult or absent adult neurogenesis the size or thickness of the DG granule cell layer was not visibly altered (Ansorg et al., 2012; Ouchi et al., 2013).

As evidenced in *Grin1*^Δ*DGCA1*^ mice, neurodegeneration following NMDAR ablation was region- and cell type-specific. Thus, dorsal but not ventral DG granule cell or CA1 pyramidal neurons were affected. A similar phenotype as the one detected in *Grin1*^Δ*DGCA1*^ and *Grin1*^Δ*DG*^ mice was also reported in two other animal models of neurodegeneration. Thus, in rats, adrenalectomy causes degeneration of granule cells in the dorsal DG. The ventral DG is much less affected, and CA1 neurons or other hippocampal regions not at all (e.g., Sloviter et al., 1993). The pro-survival effect of corticosterone appears to be mediated by mineralocorticoid receptors (Gass et al., 2000). We do not think, however, that altered downstream signaling in mice with NMDAR ablation in DG granule cell involves signaling via mineralocorticoid receptors as their expression was not affected in our mutants (data not shown).

Notably, transgenic mice overexpressing Gsk3β also exhibit degeneration of granule cells preferentially in the dorsal DG (Fuster-Matanzo et al., 2011), and the alteration is accompanied by activated astrocytes (e.g., Fuster-Matanzo et al., 2011). Interestingly, Gsk3β is more active in the dorsal, whilst Akt, an inhibitor of Gsk3β, is more active in the ventral hippocampus (Fuster-Matanzo et al., 2011). Conversely, chronic application of lithium, an inhibitor of Gsk3β, ameliorates DG neurodegeneration induced by injection of amyloid-β fibrils (De Ferrari et al., 2003). Notably, there is evidence that β-catenin, a substrate of Gsk3β, directly interacts with the NMDAR (Al-Hallaq et al., 2007). Accordingly, GluN2B-containing NMDARs suppress apoptosis induced by Gsk3β overexpression in cultured neurons (Habas et al., 2006).

It was surprising that GluN2B, but not GluN2A, was required for the survival of DG granule cells, as several studies provided ample evidence that GluN2B-containing receptors mediate excitotoxic neuronal cell death (reviewed in Hardingham and Bading, 2010). There are nevertheless reports proposing that under certain conditions GluN2B activation provides also pro-survival signals (Habas et al., 2006).

Our findings have implications for therapeutic approaches in which chronic NMDAR antagonist treatment is envisaged. Thus, NMDAR antagonists have been considered for treatment of many neurological diseases, including cerebral ischemia, traumatic brain injury, pain, Alzheimer’s disease, Huntington’s disease, Parkinson’s disease, autism spectrum disorders, and depression (reviewed in Paoletti et al., 2013). In some cases, treatment appeared promising, at least in animal models (Paoletti et al., 2013; Patrizi et al., 2015). In humans, the NMDAR antagonist ketamine shows promising effects in patients with neuropathic pain and depression (reviewed in Niesters et al., 2014; Iadarola et al., 2015). Surveillance of the literature, however, indicates that in most studies the treatment was short, ranging from 1 day to few weeks. Importantly, ketamine, is also used for recreational purposes, and the longest period of drug intake exceeds 10 years (Liao et al., 2010). Chronic use of ketamine has long-lasting devastating effects on brain function, including working memory and episodic memory deficits (reviewed in Morgan and Curran, 2012). Based on our results, we propose that NMDAR antagonists, whilst potentially beneficial for acute treatment, bear the risk of causing DG degeneration following chronic administration, which would contribute to the worsening of cognitive functions, considering the crucial role of the DG in memory processes. It is not clear, however, to which extent the results that we obtained in mice following genetic receptor ablation can be transferred to humans, and in particular whether chronic NMDAR antagonist administration mimics the results reported here. Finally, the results warrant further investigations as to the downstream cellular signal cascade that eventually leads to the region-specific cell death. It is tempting to propose a putative involvement of Gsk3β, but so far this hypothesis remains speculative.

In summary, mature granule cells in the dorsal DG undergo neurodegeneration following NMDAR ablation in aged mice. The study provides evidence that caution must be exerted, especially in aged patients, when long-term administration of NMDAR antagonists is considered for therapeutic purposes. Identification of downstream pathways leading to neurodegeneration following NMDAR ablation is warranted to eventually establish safe clinical administration of NMDAR antagonists.

## Author Contributions

YW and HM designed the experiments and wrote the manuscript. YW performed the experiments. YW and MM evaluated the data and performed the statistical analysis. All authors provided critical input for the generation of the final version of the manuscript. RS and PS provided the *Grin1*^Δ*DGCA1*^, *Grin1*^Δ*DG*^, and *Grin2a^-/-^* mice. All authors read and approved the final manuscript.

## Conflict of Interest Statement

The authors declare that the research was conducted in the absence of any commercial or financial relationships that could be construed as a potential conflict of interest.
