# Supplementary material for: Age-Dependent Degeneration of Mature Dentate Gyrus Granule Cells Following NMDA Receptor Ablation
Source: Front Mol Neurosci. 2016 Jan 12;8:87. doi: 10.3389/fnmol.2015.00087 (PMC4709453; doi:10.3389/fnmol.2015.00087)
Supplement: Supplementary file 1 [file Data_Sheet_1.DOC]

**Supplementary Material**

**Age-dependent degeneration of mature dentate gyrus granule cells following NMDA receptor ablation**

**Yasuhito Watanabe, Michaela K. Müller, Jakob von Engelhardt, Rolf Sprengel, Peter H. Seeburg, Hannah Monyer^*^**

***Correspondence:** Hannah Monyer: h.monyer@dkfz-heidelberg.de

**1 Supplementary Figures and Tables**

- 1. **Supplementary Figures**

**
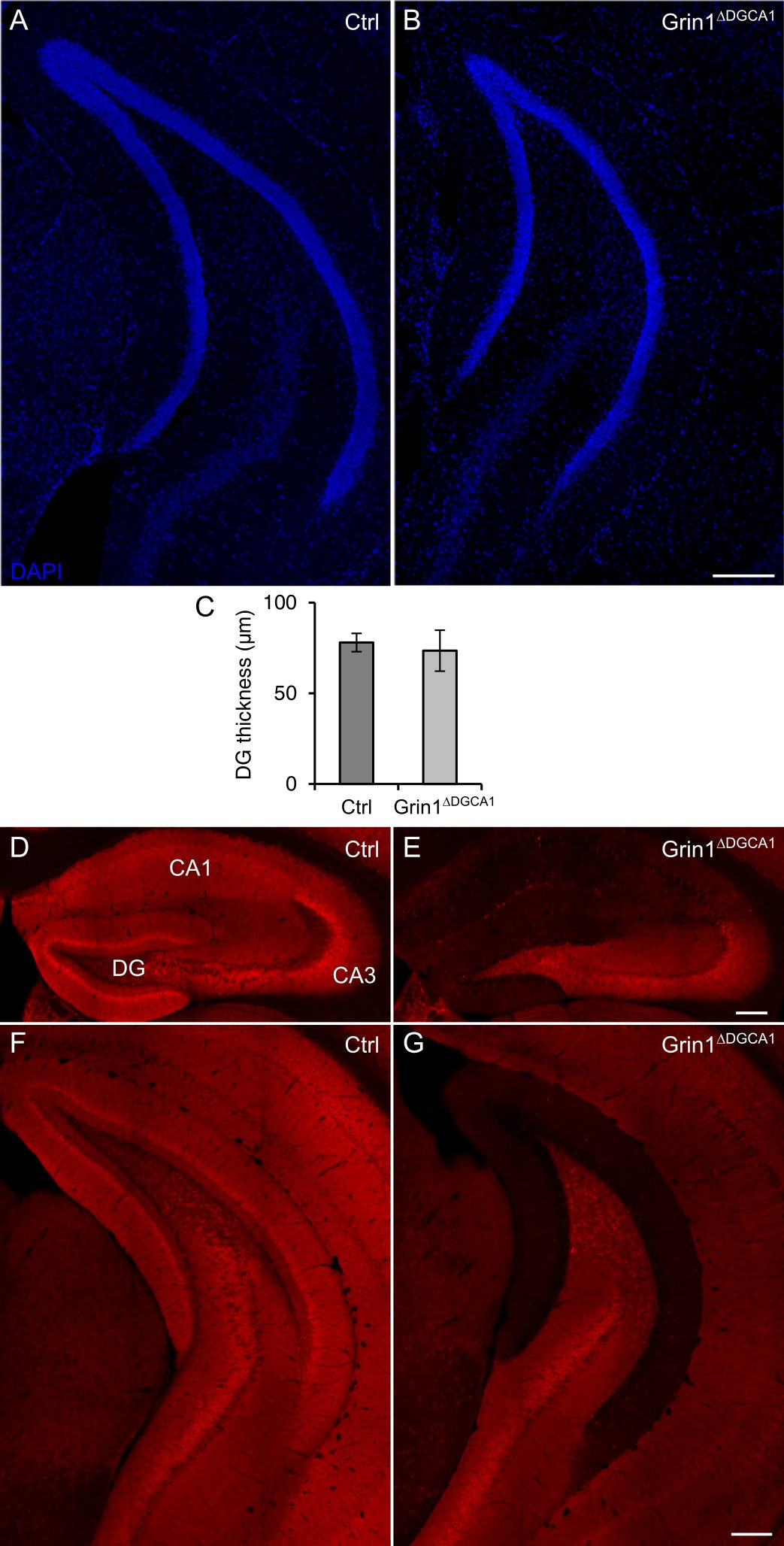
**

**Supplementary Figure 1. Ventral DG is spared in *Grin1^∆DGCA1^* mice.** **(A)** and **(B)** DAPI staining shows ventral DG from an 18 months old control (Ctrl) and *Grin1^∆DGCA1^* mouse. **(C)** Quantitative evaluation of the ventral DG granule cell layer in control and *Grin1^∆DGCA1^*mouse. Six and five mice were used for control and *Grin1^∆DGCA1^*, respectively. **(D)** GluN1 staining showing prominent expression in the whole dorsal hippocampus, including CA1 and DG in a 6 months old control mouse. **(E)** GluN1 staining in the dorsal hippocampus showing selective absence of GluN1 expression in CA1 and DG of a 6 months old *Grin1^∆DGCA1^* mouse. **(F)** GluN1 staining showing prominent expression in the ventral DG in a 6 months old control mouse. **(G)** GluN1 staining showing absence of GluN1 expression in the ventral DG of a 6 months old *Grin1^∆DGCA1^* mouse. Scale bars in **(B)**, **(E)** and **(G)**, 200 μm.

**
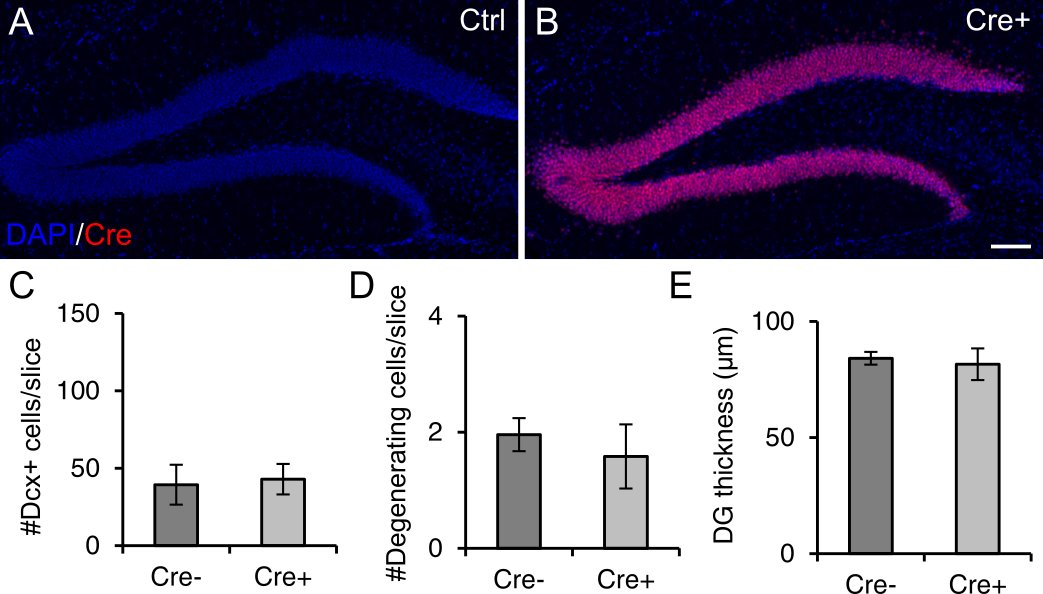
**

**Supplementary Figure 2. Cre expression per se does not induce cell death of DG granule cells.** **(A)** and **(B)** DAPI and Cre staining in a control and a Cre+ mouse, which carries both *Tg^LC1^* and *Tg^CN12^* transgenes, at the age of 6 months. **(C)** Quantitative evaluation of the DCX-positive cells in control and Cre+ mice (Student's t-test, ns). **(D)** Quantitative evaluation of the degenerating cells in control and Cre+ mice (Welch's t-test, ns). **(E)** Quantitative evaluation of the thickness of the DG in control and Cre+ mice (Welch's t-test, ns). Scale bar in **(B)**, 100 μm. Four mice were used for both the Cre- and Cre+.


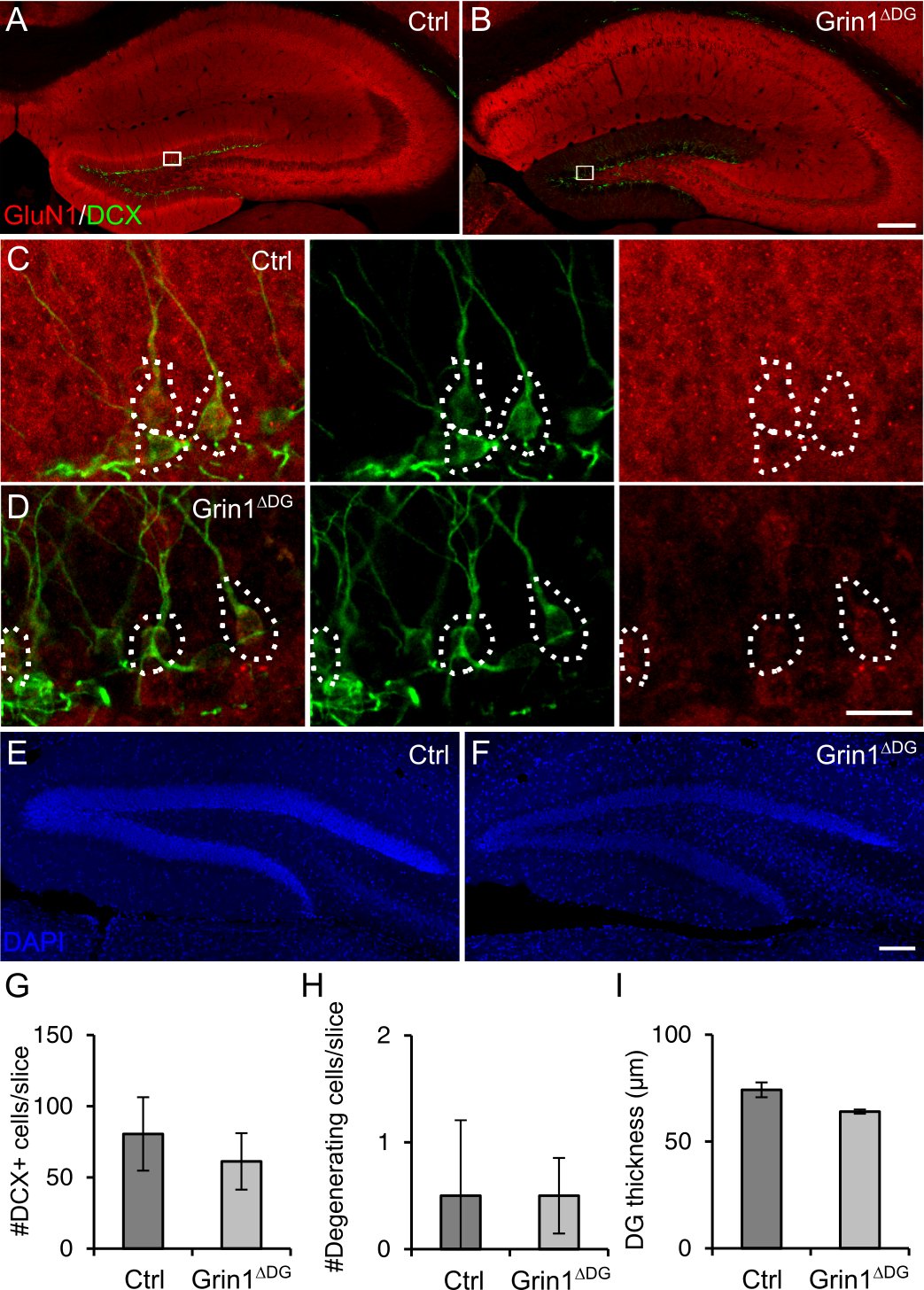


**Supplementary Figure 3. The DG of 2 months old *Grin1^∆DG^* mice is comparable to that of control mice.** **(A)** GluN1 staining showing prominent expression in the whole hippocampus including the DG in a 2 months old control mouse. **(B)** GluN1 staining in the hippocampus showing selective absence of GluN1 expression in the DG of a 2 months old *Grin1^∆DG^* mouse. **(C)** and **(D)** Magnified views of the marked areas in the DG (white boxes in **(A)** and **(B)**) show that immature neurons expressing DCX (green in **(A-D)**) retain GluN1 signals (red in **(A-D)**) in *Grin1^∆DG^* mouse **(D)**. **(E)** and **(F)** DAPI stained section showing the DG of a 2 months old control and *Grin1^∆DG^* mouse. **(G-I)** Quantitative evaluation of the number of DCX-positive cells **(G)**, the number of degenerating cells **(H)**, and the thickness of the DG **(I)** in control and *Grin1^∆DG^* mice. Scale bars in **(B)**, **(D)** and **(F)**, 200, 10 and 100 μm, respectively. Two mice were used for both control and *Grin1^∆DG^*.


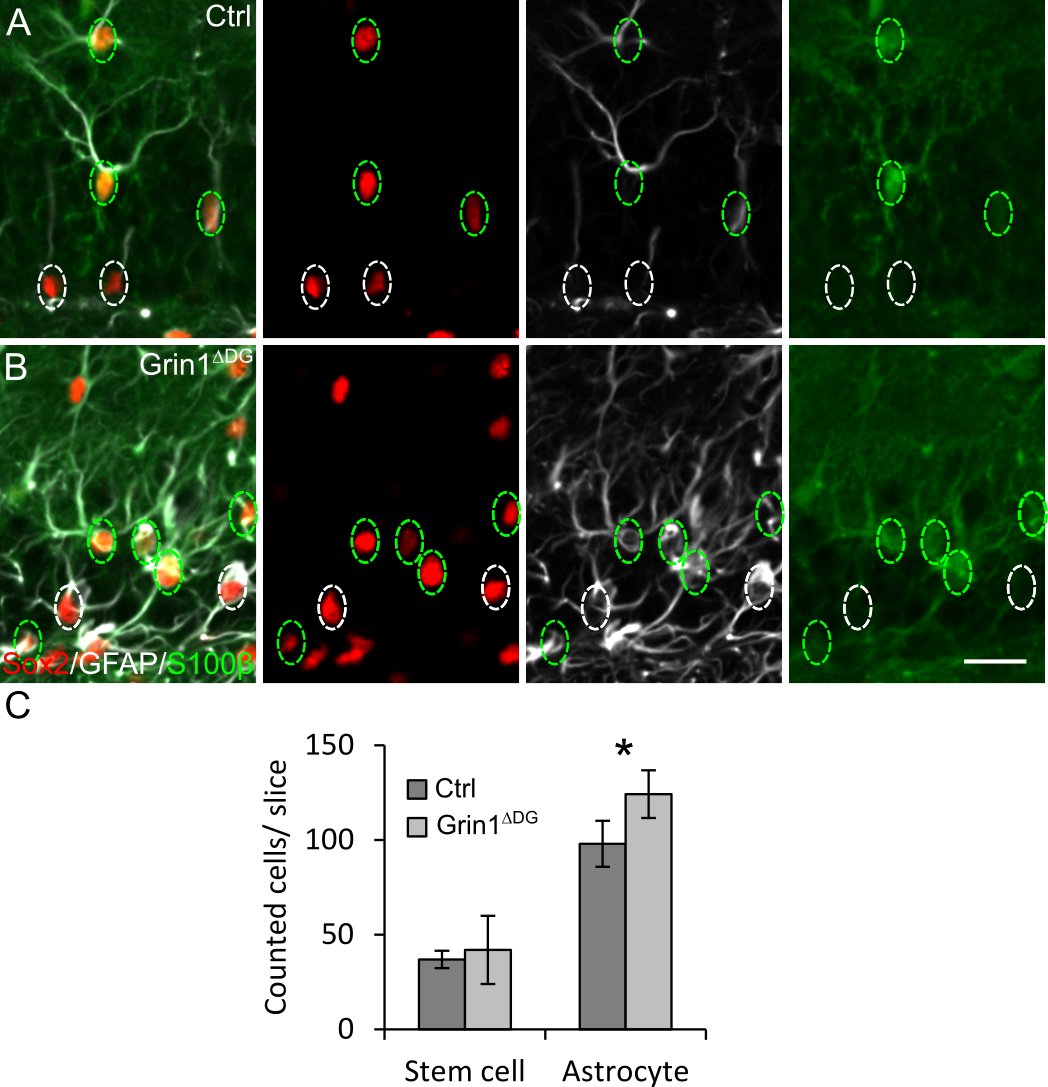


**Supplementary Figure 4. Stem cell number is not altered in *Grin1^∆DG^* mice.** **(A)** Representative section of the DG from a control mouse with labeled astrocytes (Sox2+ (red), GFAP+ (white), S100β+ (green)), and stem cells (Sox2+, GFAP+, S100β-). **(B)** Representative section of the DG from a *Grin1^∆DG^* mouse with labeled astrocytes (Sox2+ (red), GFAP+ (white), S100β+ (green)), and stem cells (Sox2+, GFAP+, S100β-). **(C)** Quantitative evaluation of astrocytes, and stem cells in the DG of control and *Grin1^∆DG^* mice (Welch's t-test with p-value modification by Holm's method, **p <* 0.05). Scale bar in **(B)**, 20 μm. The same animals were used as in Figure 3 (five control mice, and seven *Grin1^∆DG^* mice).
